# Study on the Extent of the Maillard Reaction in Chocolate

**DOI:** 10.1021/acs.jafc.5c06248

**Published:** 2025-09-02

**Authors:** Thirumal Sundaresan, P. Srinivasa Rao, Michael Hellwig

**Affiliations:** † Chair of Special Food Chemistry, Technische Universität Dresden, D-01062 Dresden, Germany; ‡ Department of Agricultural and Food Engineering, 30133 Indian Institute of Technology Kharagpur, 721302 Kharagpur, India; § Institute of Food Chemistry, Technische Universität Braunschweig, Schleinitzstraße 20, D-38106 Braunschweig, Germany

**Keywords:** chocolate, maillard reaction, glycation, caramelization, conching, furosine, *N*-ε-fructosyllysine, *N*-ε-lactulosyllysine, pyrraline, CML

## Abstract

During chocolate production, thermal processes such as roasting and conching promote nonenzymatic browning reactions such as the Maillard reaction and caramelization. In the present work, the MRPs furosine, 3-deoxyglucosone (3-DG), 3-deoxygalactosone (3-DGal), 5-hydroxymethylfurfural (HMF), *N*-ε-fructosyllysine, *N*-ε-lactulosyllysine, *N*-ε-carboxymethyllysine (CML), *N*-ε-carboxyethyllysine (CEL), pyrraline, methylglyoxal-derived hydroimidazolone 1 (MG-H1), formyline, maltosine, and rhamnolysine were quantitated in 4 filled, 12 dark, 11 milk, and 4 white chocolate samples. The predominant MRP in filled chocolates was *N*-ε-fructosyllysine (up to 2662 mg/kg of chocolate), whereas in milk chocolates, it was *N*-ε-lactulosyllysine (up to 883 mg/kg of chocolate). Filled and milk chocolates contain higher levels of furosine and CML. Dark and white chocolates exhibit lower levels of MRPs such as furosine, CML, CEL, and formyline. The consumption of milk chocolates and filled chocolates can contribute significantly to the dietary intake of pyrraline, *N*-ε-fructosyllysine, *N*-ε-lactulosyllysine, and CML.

## Graphical abstract

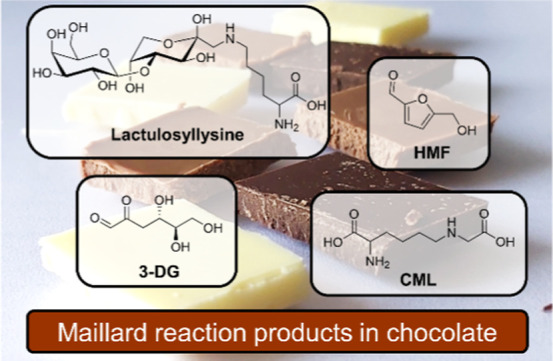

## Introduction

Chocolate is prepared from roasted and ground cocoa beans combined with powdered sugar and fat (such as cocoa butter). The three primary varieties of chocolates are dark chocolate (without milk or milk solids), white chocolate (without cocoa solids), and milk chocolate (with fewer cocoa solids and more milk solids).^1^ The production of cocoa includes fermentation, drying, roasting, and grinding of cocoa beans to obtain cocoa mass. Chocolate production, on the other hand, begins with combining cocoa mass with other ingredients (milk solids, emulsifiers, sugar, and cocoa butter), followed by refining, conching, and tempering to achieve the final product.^2^ The intake of chocolate is unevenly distributed in the world: In Switzerland, 11.8 kg per capita were consumed in 2022, 9.0 kg per capita in the US, but only 1.0 kg per capita in India and 0.1 kg in China.^3^ This is equivalent to a daily intake of up to 32 g of chocolate in Switzerland.

During food processing, many individual reactions can take place between food constituents. Browning reactions in cocoa and chocolate are linked to protein-polyphenol and Maillard reactions. The Maillard reaction, also known as “glycation”, is a series of multiple chemical reactions that take place between amino acids and reducing sugars, including lactose, glucose, and fructose. Traditionally, it is separated into three phases.^4^ It begins with the nucleophilic attack of an amino compound on the carbonyl group of a reducing sugar. The first stable reaction products are Amadori rearrangement products (ARPs). Especially the side-chain of protein-bound lysine is quite prone to glycation. ARPs break down into 1,2-dicarbonyl compounds such as 3-deoxyglucosone (3-DG), glyoxal (GO) and methylglyoxal (MGO) in the second stage of the Maillard reaction.^5^ In parallel to the reactions involving amino compounds, the process known as caramelisation allows for the direct formation of the same dicarbonyl compounds from reducing sugars at higher temperatures and lower water activity.^5^ Dehydration of 3-DG yields HMF (Figure 1). The final stage involves 1,2-dicarbonyl compounds reacting with amino and guanidino groups once more to give rise to glycated amino acids such as CEL, CML, formyline, maltosine, MG-H1, and pyrraline and then the melanoidins, which are the colored end products of the reaction. Glycation compounds are sometimes regarded as process contaminants, but their possible positive or negative effects on health are still controversially discussed.^6^

**Figure 1:**
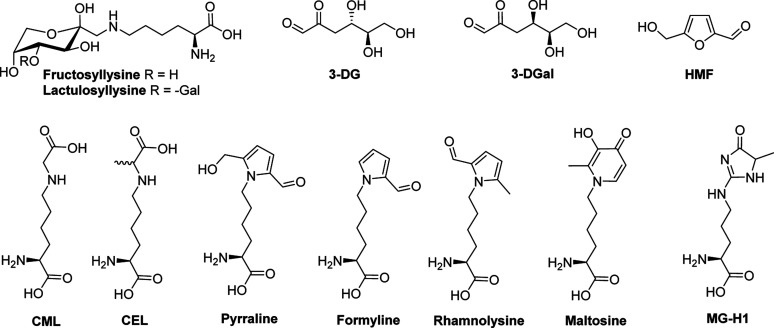
Structures of the Maillard reaction products analyzed in this study. 3-DG, 3-deoxyglucosone; 3-DGal, 3-deoxygalactosone; CEL, *N*-ε-carboxyethyllysine; CML, *N*-ε-carboxymethyllysine; MG-H1, methylglyoxal-derived hydroimidazolone 1.

One of the ARPs, fructosyl asparagine, demonstrated a prebiotic effect for *Salmonella*,^7^ which may unfavorably aid *Salmonella* in colonising the human intestine. Prior research demonstrated that ARPs can be at substantial levels in chocolate (up to 314 ± 110 mg/100 g fructosyllysine), but no differentiation for individual ARPs (e.g., fructosyllysine, lactulosyllysine) was made.^8^ The presence and concentration of ARPs such as fructosyllysine, fructosylisoleucine, fructosylmethionine, and fructosylphenylalanine in roasted and fermented cocoa samples were reported.^9^ An LC–MS/MS method with stable isotope dilution assay was developed for quantitating ARPs, revealing high concentrations in unroasted cocoa and dried bell pepper.^10^ In previous studies,^11,12^ dicarbonyl compounds such as MGO, GO, and 3-DG were quantitated in various types of chocolates such as milk, dark, and dark with high cocoa solid content (85%). The furosine levels of milk and white chocolates were quantitated as 0.64 g/kg and 0.59 g/kg, respectively.

The concentration of glycation products in chocolate may be directly influenced by differences in the ingredients, differences in processing techniques such as heating conditions during conching, and different storage conditions. Especially the presence of lactose in milk chocolate may result in an intensification of caramelization and glycation reactions. Specific glycation products such as lactulosyllysine, a disaccharide Amadori compound, may be formed. However, currently no data are available on the occurrence or concentration of these compounds in different varieties of chocolates, especially milk chocolate.

This study aimed first to provide an overview of the extent of glycation products in chocolate, and second to estimate the contribution of chocolate consumption to the dietary intake of glycation compounds. Therefore, original data on the amount of furosine in various chocolate products, as well as the individual ARPs that make up furosine were obtained. Additionally, it intended to provide insights into other MRPs of the advanced (dicarbonyl compounds, HMF) and late stages (CML, formyline, and pyrraline).

## Materials and Methods

### Chemicals

Prolidase (553 U/mg protein), quinoxaline, petroleum ether, acetic acid, pepsin (3839 U/mg protein), sodium dihydrogen phosphate, sodium borate, leucine aminopeptidase (18 U/mg protein), trifluoroacetic acid (TFA), *o*-phenylenediamine (OPD), Pronase E (4000 PU/mg), 5-hydroxymethylfurfural (HMF), and nonafluoropentanoic acid (NFPA) were all acquired from Sigma-Aldrich (Steinheim, Germany). Fisher Scientific (Loughborough, UK) provided methanol and acetonitrile of HPLC-MS/MS grade. Merck (Darmstadt, Germany) supplied zinc sulfate heptahydrate. VWR (Darmstadt, Germany) provided the HPLC gradient grade acetonitrile and hydrochloric acid. Riedel-de-Haën (Seelze, Germany) provided Tris and potassium hexacyanoferrate­(II). Water for the preparation of buffers and solutions was double distilled in-house (Destamat Bi 18E, QCS GmbH, Maintal, Germany). The standards of MRPs, including *N*-ε-fructosyllysine,^13^ formyline,^14^ *N*-ε-lactulosyllysine,^15^ rhamnolysine,^16^ CML,^15^ pyrraline,^15^ MG-H1,^15^ maltosine,^54^ CEL^15^ as well as the quinoxaline derivatives of dicarbonyl compounds^17^ were synthesized in compliance with the protocols provided in the corresponding publications.

### Chocolate Samples

Thirty-one chocolate samples were obtained from commercial providers, comprising 12 dark chocolates, 11 milk chocolates, 4 white chocolates, and 4 filled chocolates (Table S1). Fillings included milk and cocoa creams, hazelnuts and egg liqueur. All of the samples were examined directly after being obtained. First, the samples were manually grated into small pieces to ensure uniform size. For the preparation of defatted samples, 3 g of grated chocolate was mixed with 25 mL of petroleum ether in a 50 mL centrifuge tube, weighed, and shaken. After centrifugation for 10 min at 9000 g at room temperature, the petroleum ether phase was discarded. The extraction process was repeated twice. The residues were then dried overnight.

### Quantitation of Furosine

In a large hydrolysis tube, 50 mg of defatted chocolate was mixed with 10 mL of 6 M HCl, and hydrolysis was carried out for 23 h in a drying cabinet preheated to 110 °C.^18^ After cooling, the samples were filtered into glass vials and stored at 4–8 °C. For solid-phase extraction, a cartridge (Strata C-18E, 55 μm, 70 Å; Phenomenex, Aschaffenburg, Germany) was conditioned with 5 mL of methanol followed by 10 mL of water. Then, 500 μL of acid hydrolysate was applied, and elution was performed with 3 mL of 3 M HCl in increments (500 μL + 500 μL + 1000 μL + 1000 μL). The complete eluate was evaporated to dryness using a water jet pump, and the residue was quantitatively dissolved in 1 mL of 0.1% aqueous trifluoroacetic acid (TFA).

All chromatographic works were conducted using a low-pressure gradient system (Elite LaChrom, Hitachi, Germany) including an online degasser, a diode array detector (DAD) (L-2455), a column oven (L-2350), a pump (L-2130), and an autosampler (L-2200). Spectra were recorded during chromatographic runs from 190 to 400 nm (increment of 1 nm). As eluents, 0.1% TFA (solvent A) and 90% acetonitrile in distilled water (solvent B) were employed, along with a column (Eurospher II–C18, 250 mm × 4.6 mm, 5 μm, 100 Å; Knauer, Berlin, Germany) at 33 °C. A gradient was applied as follows: 5% solvent B at 2 min, 50% B at 25 min, 50% B at 27 min, 5% B at 30 min, and 5% B at 35 min, at a flow rate of 0.6 mL/min. A furosine standard that had been independently synthesized was used for all calibrations.

### Quantitation of 1,2-Dicarbonyl Compounds

In accordance with a previously reported technique,^19^ defatted chocolate samples were extracted with water, proteins were precipitated with methanol, and the extracts were derivatized with OPD to analyze 1,2-dicarbonyl compounds. The HPLC system was calibrated using quinoxaline standards synthesized from 3-DG, 3-DGal, and 3-deoxypentosone (3-DPs),^17^ as well as commercially available quinoxaline (the GO derivative) and 2-methylquinoxaline (the MGO derivative).

### Ultrahigh-Performance Liquid Chromatography with Time-of-Flight Mass Spectrometric Detection (UHPLC-TOF-MS)

Selected OPD-derivatized samples were filtered (0.2 μm), and 1 μL of the samples was injected into a UHPLC system (Infinity 1290, Agilent). Solvent A was 0.2% acetic acid in MS-grade water, and solvent B was MS-grade methanol. An RP column (Zorbax Eclipse Plus, 50 × 2.1 mm, 1.8 μm, Agilent) was used at a column temperature of 25 °C in the gradient mode (0 min, 5% B; 25 min, 90% B; 30 min, 90% B; 31 min, 5% B; 37 min, 5% B) at a flow rate of 0.2 mL/min. The absorption was recorded at the wavelengths 280 and 312 nm. The HPLC system was connected to the high-resolution mass spectrometer TIMS-TOF (Bruker Daltonics, Bremen, Germany), working in the positive scan mode (*m*/*z* 20–1300; scan time, 500 ms; dry gas flow, 10 L nitrogen/min; dry temperature, 220 °C; nebulizer pressure, 2.2 bar; capillary voltage, 4500 V).

### Quantitation of 5-Hydroxymethylfurfural

Defatted chocolate samples (250 mg) were incubated for 30 min at 30 °C in a water bath with 1000 μL of 0.1 M sodium borate buffer (pH 8.2).^19^ The samples were centrifuged for 10 min at 9000 g at room temperature after shaking (2 min). Carrez I solution (30 μL, 15% (w/v) potassium hexacyanoferrate­(II) in water) and Carrez II solution (30 μL, 30% (w/v) zinc sulfate heptahydrate in water) were combined with 600 μL of the supernatants while being shaken intermittently. The mixtures were incubated at 4 °C for 1 h. After incubation, the mixtures were centrifuged for 10 min at 9000 g at room temperature before being analyzed by RP-HPLC. A low-pressure gradient system (Elite LaChrom, Hitachi, Germany), including a DAD (L-2455), a column oven (L-2350), a pump (L-2130), an online degasser, a solvent organizer, and an autosampler (L-2200), were used to analyze the mixtures. An RP column (Eurospher C-18, 250 mm × 4.6 mm, 5 μm, 100 Å; Knauer) with a guard column (5 mm × 4 mm) of the same material was employed for the separation at room temperature. At a flow rate of 1 mL/min, 5% acetonitrile in water was used as an isocratic eluent for 30 min after injection of 20 μL of the supernatants. The absorbance was measured at 280 nm while spectra (190–400 nm, increment 1 nm) were simultaneously recorded. A commercial HMF standard was used for external calibration (c = 1.7–43.2 mg/L).

### Quantitation of Glycated Amino Acids

Samples of defatted chocolate underwent a three-step enzymatic hydrolysis process.^18^ In a 4 mL vial, 20 mg of defatted chocolate was weighed, followed by the addition of 1 mL of 0.02 M HCl and 50 μL of pepsin solution (2 mg/mL in 0.02 M HCl). The samples were incubated for 24 h at 37 °C in an incubation cabinet. After adding 250 μL of 2 M Tris buffer (pH 8.2) and 50 μL of Pronase E solution (2 mg/mL), the samples were incubated for an additional 24 h at 37 °C. Following the addition of 0.4 U (4 μL) of aminopeptidase solution and 1 U (10 μL) of prolidase solution, the samples were incubated for a final 24 h at 37 °C. After incubation, the samples were employed for HPLC-MS/MS analysis.

An HPLC-MS/MS system comprising a binary pump (G7104C), an autosampler (G7129C), a column thermostat (G7116 A), and a triple-quadrupole mass spectrometer Ultivo (G6465 B; all from Agilent Technologies, Böblingen, Germany) were used to quantitate glycated amino acids. Capillary voltage was 4000 V, and the nebulizing gas was nitrogen (gas flow, 13 L/min; gas temperature, 300 °C; nebulizer pressure, 35 psi). For separations, a Zorbax Eclipse Plus C18 column (2.1 × 100 mm, 3.5 μm; Agilent) was utilized. The HPLC solvents A and B were 10 mM nonafluoropentanoic acid (NFPA) in water and 10 mM NFPA in acetonitrile, respectively. A flow rate of 0.25 mL/min was used to pump the solvents at a column temperature of 35 °C (0 min, 2% B; 7 min, 5% B; 10 min, 30% B; 17 min, 60% B; 19 min, 2% B; 24 min, 2% B). The injection volume was 5 μL.

Prior to analysis, all enzymatic hydrolysates were diluted by combining 100 μL of the enzymatic hydrolysate with 900 μL of 10 mM NFPA, followed by centrifugation at 9000*g* for 10 min. Quantitation was performed by matrix calibration, taking two samples of milk, dark and white chocolates. Diluted samples (100 μL) were mixed with increasing volumes of a standard mix. Linearity of the calibration curves is shown in Figure S1. Prior to injection, every sample was centrifuged at 9000 g for 10 min at room temperature. Subsequently, 80 μL of the supernatant was used for LC–MS analysis. Table 1 compiles the MRM measurement operating conditions. The standard mix contained *N*-ε-fructosyllysine (5.7 μg/mL), *N*-ε-lactulosyllysine (58.8 μg/mL), *N*-ε-carboxymethyllysine (0.14 μg/mL), *N*-ε-carboxyethyllysine (0.13 μg/mL), pyrraline (0.89 μg/mL), formyline (0.13 μg/mL), MG-H1 (0.17 μg/mL), maltosine (55.4 ng/mL), and rhamnolysine (66.2 ng/mL).

**Table 1:** Transitions Recorded During MRM Measurement of Glycated Amino Acids

compound	precursor ion [*m*/*z*]	product ion [*m*/*z*]	fragmentor voltage [V]	collision energy [eV]	Q/q[^a^]
lactulosyllysine	471	128	140	20	q
	471	225	140	20	Q
fructosyllysine	309	84	120	30	Q
	309	225	120	10	q
pyrraline	255	148	80	20	q
	255	175	80	10	Q
maltosine	255	84	120	20	Q
	255	126	120	10	q
rhamnolysine	239	175	100	12	q
	239	148	100	24	Q
MG-H1	229	114	120	10	Q
	229	166	120	10	q
formyline	225	134	80	20	Q
	225	161	80	10	q
CML	205	130	100	10	q
	205	84	100	20	Q
CEL	219	130	100	10	q
	219	84	100	20	Q

a Q, transition used for quantitation; q, transition used for the confirmation of the presence of the analyte. Dwell time, 70 ms.

The theoretical furosine content of the chocolates was estimated from the contents of fructosyllysine (w_FL_) and lactulosyllysine (w_LL_) as follows

$$w_{Fur}=254.2\times \left(0.324\times \frac{w_{FL}}{308.2}+0.338\times \frac{w_{LL}}{470.2}\right)$$

with 254.2, 308.2, and 470.2 as the molecular masses of furosine, fructosyllysine, and lactulosyllysine, and 0.324 and 0.338 as the molar conversion factors of these Amadori products in 6 M HCl.^13^

### Statistical Analysis

Substances were regarded as “not determined (i.e., < LOD)” when their peaks exhibited signal-to-noise ratios of less than 3. Substances were regarded as to be present in “traces” when their peaks showed signal-to-noise ratios between 3 and 10 (i.e., < LOQ). All analyses were done in duplicate. Correlation analysis was performed with the software PASW Statistics 18, and Spearman’s rank correlation coefficients (*r*_S_) and significance of correlations were calculated.

## Results and Discussion

### Quantitation of Furosine

The present study was carried out to gather information on the extent of the Maillard reaction in chocolate and to estimate the contribution of chocolate consumption to the dietary intake of glycation compounds. First, furosine was determined in different types of chocolates, such as dark, milk, or white chocolates and chocolates with filling. Furosine determination is an indirect method of measuring the total amount of Amadori compounds (e.g., *N*-ε-fructosyllysine, *N*-ε-lactulosyllysine) generated by the interaction of the ε-amino group of lysine with reducing sugars (e.g., glucose, lactose) in food and is used as a marker of heat damage to food proteins.^20^ The chromatogram of a chocolate sample acquired by RP-HPLC with the C-18 column is displayed in Figure S2. Furosine was identified in all acid-hydrolyzed chocolate samples by comparing retention time and the distinctive UV spectrum with its absorption maximum at 280 nm, as in previous works.^18,21^

Chocolates with filling and milk chocolates had the highest furosine concentrations (369–714 mg/kg; 53.3–421 mg/kg). In contrast, all tested white and dark chocolate samples (55–121 mg/kg; 7.4–103 mg/kg) had substantially lower furosine levels (Table 2, Figure S3). The high amount of furosine in chocolates with filling and milk chocolate is supposedly due to the reducing sugar lactose gaining higher importance in milk solids-added samples. The comparatively high content of protein in filled and milk chocolates (8.6–9%; 5.5–7.9%) promotes the formation of ARPs during the initial stage of the Maillard reaction. In contrast, dark chocolate tended to have lower furosine concentrations because it contains less sugar (12.6–49%) and mainly nonreducing sucrose as compared to milk chocolate (47–59% sugar), which also contains lactose. White chocolate also showed lower furosine due to less or absence of cocoa solids, because this reduces the protein content (4.2–6.4% in white chocolates without filling) of chocolate. The furosine levels that were analyzed in the present study are consistent with those of previous research, where furosine had been expressed as fructosyllysine. A fructosyllysine content of 3.14 ± 1.10 g/kg in several chocolates was reported, equivalent to 830 ± 290 mg/kg furosine.^8^

**Table 2:** Concentrations of Individual Maillard Reaction Products in Different Chocolate Samples

MRPs	types of chocolate			
	filled^a^ (*n* = 4)	dark^a^ (*n* = 12)	milk^a^ (*n* = 11)	white^a^ (*n* = 4)
furosine	557 (369–714)	26.6 (7.4–103)	169 (53.3–421)	88 (55–121)
3-DG	155 (76–299)	18.2 (10.4–28.6)	7.4 (5.2–9.4)	4.6 (1.6–6.9)
3-DGal	6.8 (4.6–8.9)	2.8 (2.3–4.3)	2.1 (0.57–3.9)	1.8 (0.28–2.8)
HMF	27 (14.5–46.8)	1.2 (0.37–4.8)	0.63 (0.14–1.4)	0.05 (0.03–0.06)
CEL	2.7 (2–3.4)	2.1 (1.7–3.2)	0.87 (nd-2)	0.59 (nd-1)
CML	47.2 (24.2–64.8)	6.6 (4.3–18.1)	18.9 (8.3–108)	9.8 (6.3–13.5)
formyline	2.4 (1.7–2.9)	2.5 (1.5–4.5)	1.4 (tr-3.7)	1.1 (tr-1.1)
*N*-ε-fructosyllysine	2662 (969–5337)	20.9 (7.3–57.5)	40.7 (19.2–59)	123 (116–156)
*N*-ε-lactulosyllysine	787 (313–843)	18.5 (nd-736)	883 (184–3170)	649 (496–1637)
maltosine	nd	1.5 (nd-1.9)	nd-tr	nd
MGH1	7.7 (5.9–8.9)	6.8 (tr-9.1)	15.3 (nd-15.8)	nd
pyrraline	40.8 (27.3–54.3)	33.6 (21.9–44.4)	13.5 (9–48)	2.8 (2.5–3.3)
rhamnolysine	nd	2.4 (nd-2.7)	nd	nd

a Data are given in mg/kg of chocolate, based on the median levels and the ranges given in parentheses. nd, not detectable, below LOQ; tr, traces, between LOD and LOQ.

Due to their high lysine content, milk proteins are extremely sensitive to the Maillard reaction, which causes the ARP *N*-ε-lactulosyllysine to develop quickly under mild heating conditions. Furthermore, considering that dried milk may contain significant levels of furosine,^22^ some of the furosine measured in milk, white, and chocolates with filling may have been carried over from the ingredients. The furosine content in dark chocolate is attributed to its high cocoa solids and the Maillard reaction products^23^ formed during the fermentation of cocoa beans, as it reduces the moisture content and provides the optimum condition for the production of ARPs during the chocolate manufacturing process. This study aligns with studies conducted on different biscuit samples that found that the samples with high sugar and protein content exhibited high amounts of furosine.^24^ The changes in the reducing sugar and protein levels in the flours utilized can be correlated to the variations in furosine concentration among different breads.^25^ In another study, the furosine content was analyzed in milk and white chocolate after hydrolysis with 6 M HCl, showing that furosine levels were higher in milk chocolate (640 mg/kg) compared to white chocolate (590 mg/kg).^12^

### Quantitation of Dicarbonyl Compounds

The presence of 1,2-dicarbonyl compounds in food can reveal information about processing methods, storage conditions, and the degree of heat exposure.^26^ In chocolate samples, 3-DG and 3-DGal were identified as major dicarbonyl compounds, and others such as MGO, GO, and 3-DPs were not detected (Figure S4). The different reactivity of these compounds can explain this disparity. Because of their greater reactivity, GO and MGO are more likely to have more extensive reactions during heat treatment,^27^ which could result in lower concentrations that are undetectable and, consequently, higher development of glycation compounds derived from them, as was also seen in this investigation. Conversely, 3-DG could accumulate in the final product before undergoing degradation reactions. The concentration of 3-DG in chocolate samples was notably higher than that of 3-DGal. The formation of 3-DGal from 3-DG through epimerization is a plausible explanation for these results.^26^ 3-DG and 3-DGal were present in high concentrations in chocolates with filling (76–299 mg/kg; 4.6–8.9 mg/kg), followed by dark (10.4–28.6 mg/kg; 2.3–4.3 mg/kg), milk (5.2–9.4 mg/kg; 0.57–3.9 mg/kg), and white chocolates (1.6–6.9 mg/kg; 0.28–2.8 mg/kg) (Table 2, Figure S3). Filled chocolates contained high levels of 3-DG and 3-DGal, which should have been carried over from carbohydrate- and protein-rich ingredients and the high thermal processing during chocolate manufacturing.^28^ Current findings are consistent with earlier research,^11^ where high levels of 1,2-dicarbonyl compounds (26–555 mg/kg) in sweets and chocolates were reported, with the highest levels in chocolate bars due to added ingredients such as caramel.

### Detection of Disaccharide Dicarbonyl Compounds

The derivatized dicarbonyl compounds were also analyzed by RP-HPLC with time-of-flight mass spectrometric detection to qualitatively investigate if disaccharide dicarbonyl compounds can be formed in white or milk chocolates. Besides the quinoxalines of 3-DG and 3-DGal eluting between 7.3 and 7.5 min, two further prominent UV-active peaks with retention times of 5.8 and 7.0 min were detected in a sample of milk chocolate (Figure 2). In the mass spectrum of the peak eluting at 7.0 min, a signal with an *m*/*z* ratio of 397.1606 was observed (Figure S5A). The respective extracted ion chromatogram shows one major peak at the same retention time (Figure 2B). This *m*/*z* is equivalent to a quinoxaline adduct of monodehydrated lactose as shown in Figure 2 (C_18_H_25_N_2_O_8_^+^, *m*/*z*_theo._ = 397.1605, Δ*m*/*z* = 0.3 ppm). The main fragment with *m*/*z* = 235.1077 (C_12_H_15_N_2_O_3_^+^, *m*/*z*_theo._ = 235.1077, Δ*m*/*z* < 0.5 ppm) results from the loss of the galactose residue. Using low-resolution mass spectrometry, *m*/*z* = 235 was also detected as the main fragment in the fragmentation of the quinoxaline of 3-deoxymaltosone.^18^ Further fragment ions of the structure had *m*/*z* = 199.0872 (C_12_H_11_N_2_O^+^, *m*/*z*_theo._ = 199.0866, Δ*m*/*z* = 3.0 ppm), *m*/*z* = 171.0923 (C_11_H_11_N_2_^+^, *m*/*z*_theo._ = 171.0917, Δ*m*/*z* = 3.5 ppm), *m*/*z* = 157.0755 (C_10_H_9_N_2_^+^, *m*/*z*_theo._ = 157.0760, Δ*m*/*z* = 3.2 ppm), and *m*/*z* = 145.0757 (C_9_H_9_N_2_^+^, *m*/*z*_theo._ = 145.0760, Δ*m*/*z* = 2.1 ppm). A tentative assignment of these fragments to individual structures is included in the supplement (Figure S5B). A signal with *m*/*z* = 195.0874 could not be assigned. The quinoxaline of lactosone was also detected (*m*/*z* = 413.1555).

**Figure 2:**
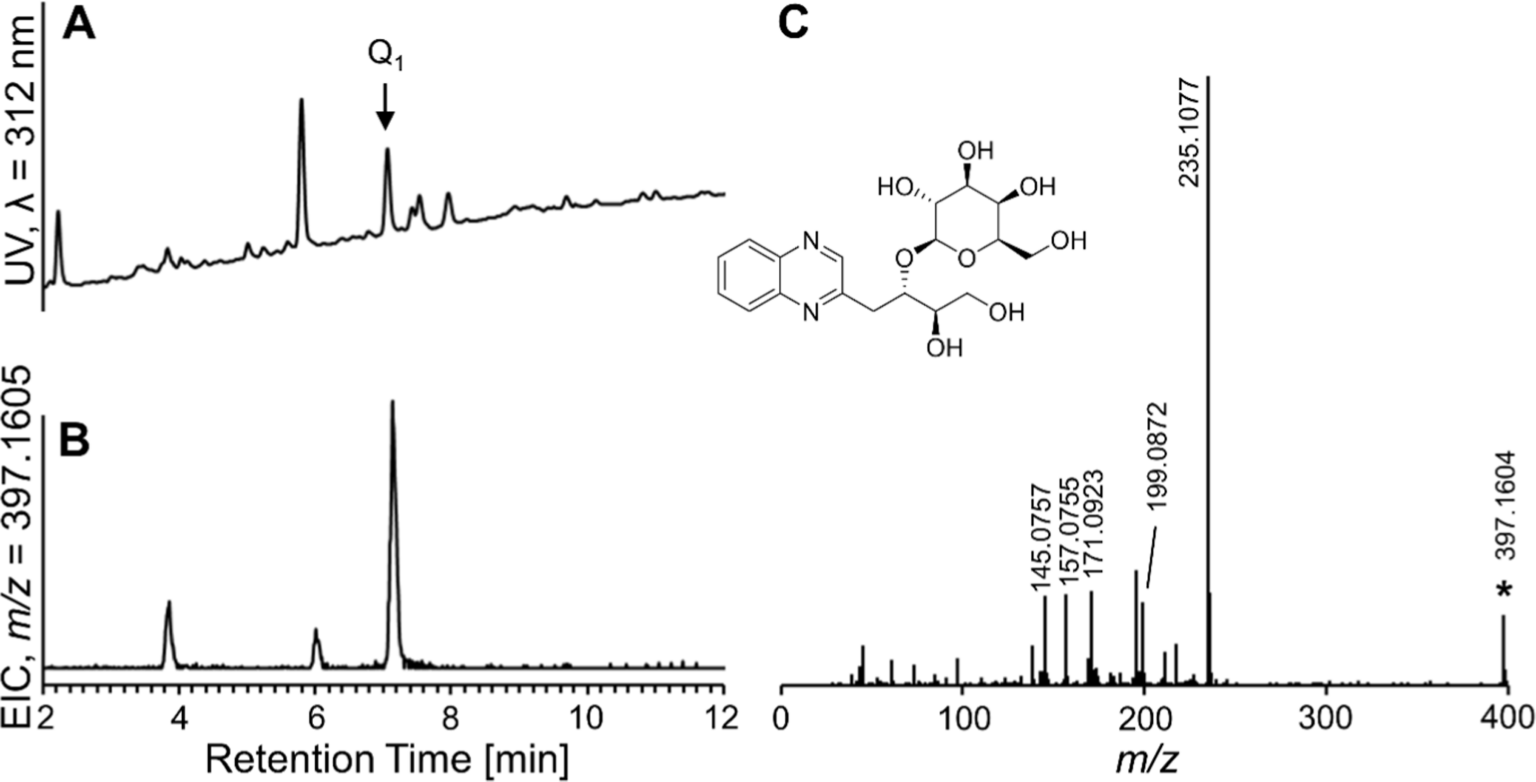
RP-HPLC with UV and mass-spectrometric detection of a defatted milk chocolate sample derivatized with *o*-phenylenediamine. (A) UV chromatogram at λ = 312 nm. (B) Extracted ion chromatogram (*m*/*z* = 397.1605). (C) MS/MS spectrum of *m*/*z* = 397.1606. The structure of the quinoxaline of 3-deoxylactosone as a possible structure of Q1 is shown in the center.

Regarding sugar degradation pathways in the literature for maltose and lactose,^18,29,30^ we suppose that the tentatively identified structure is equivalent to the quinoxaline of 3-deoxylactosone, though the reaction product of 1-deoxylactosone would also be possible. Comparison with literature data revealed that the fragment *m*/*z* = 159.0917 is absent from the fragmentation mass spectrum of the quinoxaline derived from 3-DG, while it is sometimes used as a fragment ion of methylquinoxalines in MRM methods.^31^ In the spectrum in Figure 2C, such a fragment was not found, so that it becomes less probable that the peak results from 1-deoxylactosone. Standard synthesis and characterization will be needed to unequivocally assign the compound.

### Quantitation of HMF

HMF as a dehydration product of 3-DG is generated when a sugar-rich product is heated for an extended period of time at 150 °C in an acidic environment (the pH of cocoa beans is around 5.7 before roasting). This causes sugars to caramelize, which is most readily achieved by glucose, fructose, or ribose.^32^ Moreover, HMF is formed during the degradation of ARPs in the Maillard reaction. HMF is an established tool for assessing the quality of food and is closely linked to food processing and storage.^33^

In this study, HMF had a similar trend as dicarbonyl compounds such as 3-DG and 3-DGal and the HMF content of chocolate samples varied among dark (0.37–4.8 mg/kg), milk (0.14–1.4 mg/kg), white (0.03–0.06 mg/kg), and chocolates with filling (14.5–46.8 mg/kg) (Table 2, Figures S3 and S6). The concentrations of HMF and 3-DG also correlated significantly (Table S3). Chocolates with filling exhibited the highest amount of HMF, either due to carry-over of HMF itself or the monosaccharide precursors from ingredients such as added sugar, whole milk powder, glucose syrup, dextrose and condensed skimmed milk. According to the literature, the higher carbohydrate content, lower moisture content and the addition of glucose syrup increases the HMF content as it acts as the main precursor of furanic compounds.^34^ A direct correlation between the sugar content in a recipe and the HMF levels in baked cookies was reported.^35^

This study found that dark chocolate has a significantly higher amount of HMF than milk chocolate. Dark chocolate samples with a higher content of cocoa solids (>70%) showed significantly higher levels of HMF probably because of carry-over from the cocoa solids. The high HMF content in cocoa solids was already described in the literature and increases during roasting.^36^ The impact of different roasting conditions, such as temperature and time, on the stability of Amadori compounds and their direct influence on the formation of downstream Maillard reaction products like pyrazines and furans (e.g., 5-methylfurfural) has been investigated in the literature using fructosylalanine. While this compound remains stable for at least 60 min at 140 °C, it is almost completely degraded after only 5 min at 200 °C.^37^ The roasting conditions can even be controlled in such a way that the minimization and maximization of individual reactions becomes possible.^38^ Samples with a lower content of cocoa solids (46–50%) exhibited less HMF content compared to other dark chocolate samples. Some of the added ingredients in dark chocolate like cane sugar, ground vanilla pods, low-fat cocoa powder, skimmed milk powder,^39^ and sweet whey powder also may have contributed significantly to the HMF content in the dark chocolates.

The milk chocolate samples with higher protein content (>5.8 g/100 g) from added cocoa mass, vanilla pods, and hazelnut paste and higher sugar content (55 g/100 g) may carry-over HMF from added whole milk powder,^40^ sweet whey powder, and skimmed milk powder, as they were found to have a significantly higher HMF content than other milk chocolate samples, corresponding with the literature.^41^ However, the lower HMF content in milk chocolates compared to chocolates with filling and dark chocolates might be attributed to the presence of a lower amount of cocoa solids (<40%). Most of the white chocolate samples contained significantly lower amounts of HMF when compared to other chocolate samples, as no cocoa solids were added. This study is in accordance with an earlier study on the occurrence of HMF in chocolates/pralines, where a mean HMF concentration of 273.8 mg/kg was determined.^42^

The heat-induced processes like drying in cocoa production increase HMF levels,^43^ while other studies^32,35^ emphasize that fermentation and conching processes significantly impact the final HMF concentration. The HMF content of chocolates in this study was lower than that obtained in previous research on different varieties of chocolate samples (42–164 mg/kg).^43^ This discrepancy may be attributed to different ingredients, variations in production, and manufacturing conditions.

### Quantitation of Glycated Amino Acids

In food products that have undergone more intense heat treatment, where ARPs may already undergo degradation, glycated amino acids may provide further information on the degree of the Maillard reaction. Lysine-derived compounds, CML and CEL, are utilized as markers to evaluate the extent of heat damage and protein modification.^44^

The chromatographic method chosen for these analyses permitted the separation of lactulosyllysine and fructosyllysine (Figure 3). In this work, it was determined that the main ARP in milk chocolate was *N*-ε-lactulosyllysine, with higher concentrations than in other chocolate types. This can be due to the reaction of lactose on cocoa and milk proteins during production, but also to a carry-over of preformed lactulosyllysine in whole milk powder and skim milk powder.^45^ In contrast to white, milk, and dark chocolates, chocolates with filling showed much higher levels of *N*-ε-fructosyllysine. This may be attributed to the carry-over of glucose from ingredients such as glucose syrup, dextrose, and other added sugars.^46^ The median levels of *N*-ε-lactulosyllysine in our research were 883 mg/kg in milk, 787 mg/kg in filled, 649 mg/kg in white, and 18.5 mg/kg in dark chocolates. However, though HPLC-MS/MS with MRM detection is a very specific method, it may reach its limitations when it comes to isobaric compounds. Hence, in the dark chocolates, the identity of lactulosyllysine is not sure, and it might just as well be maltulosyllysine. All reducing disaccharides can form a disaccharide ARP with the same mass and eventually similar fragmentation as lactulosyllysine. In a previous study, the same transitions as used here for lactulosyllysine were chosen for maltulosyllysine.^18^ Meanwhile, the median levels of *N*-ε-fructosyllysine were 2662 mg/kg in filled, 123 mg/kg in white, 40.7 mg/kg in milk, and 20.9 mg/kg in dark chocolates. Furosine was determined by acid hydrolysis to determine the extent to which the amounts of *N*-ε-fructosyllysine and *N*-ε-lactulosyllysine in chocolate may account for the total Amadori compounds-derived lysine modification. Acid hydrolysis produces furosine from ARPs in specific molar yields.^13^ In dark chocolates, furosine levels were generally low, but they were about three times higher than theoretical furosine, possibly due to other Amadori products present in the sample that give furosine during acid hydrolysis, e.g., resulting from oligosaccharides. Milk chocolates showed excellent conversion of Amadori products to furosine content by 90%. In white chocolates, only 50% of the theoretical furosine was actually found as furosine, likely due to matrix effects hindering furosine formation during hydrolysis.

**Figure 3:**
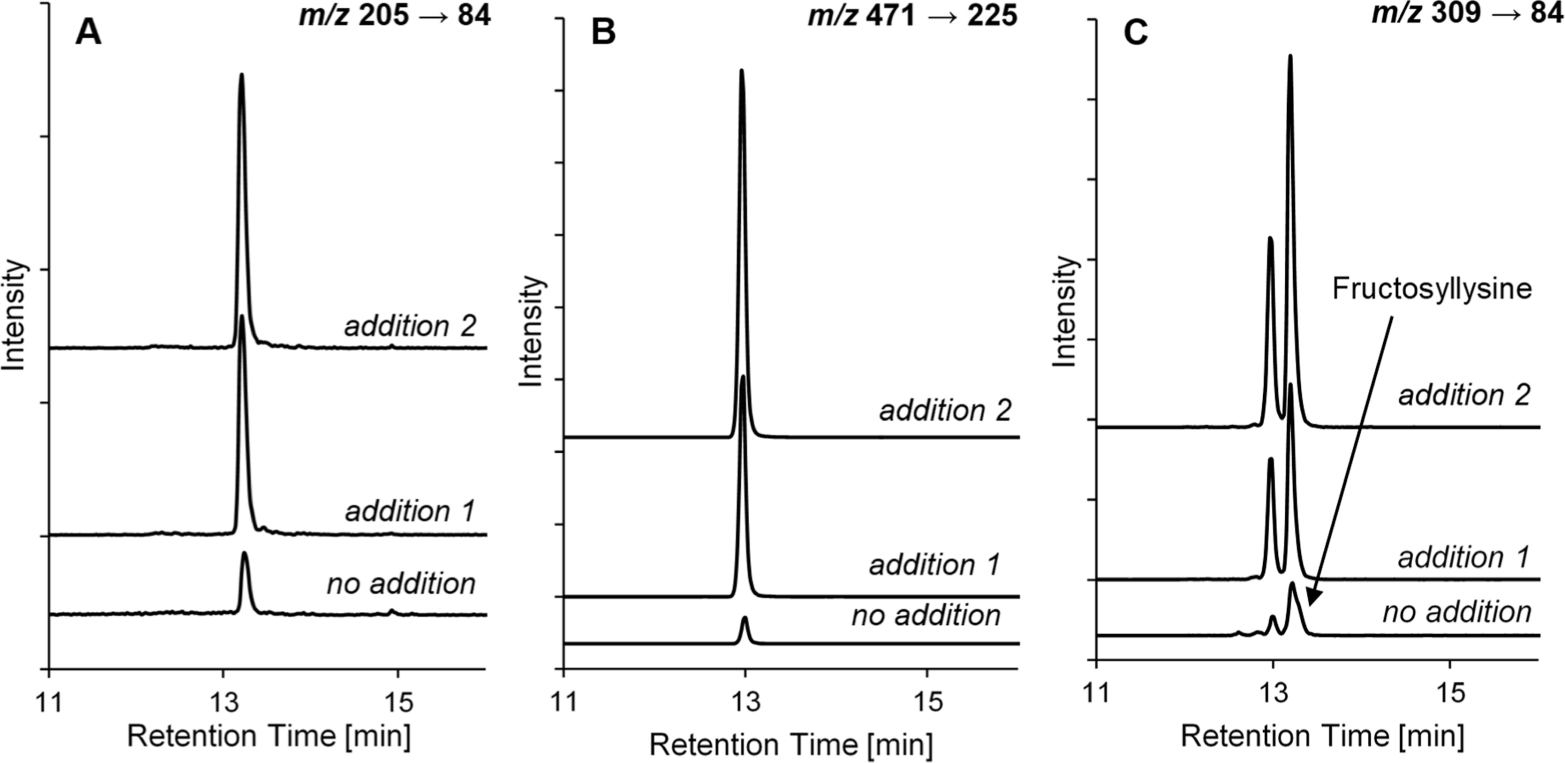
RP-HPLC-MS/MS (MRM mode) of an enzymatically hydrolyzed chocolate sample without and with (A) CML, (B) lactulosyllysine, and (C) fructosyllysine standard added.

After the ARPs, CML was the most abundant compound in filled and milk chocolates (Figure 3), with concentrations ranging from 24.2 to 64.8 mg/kg in filled and 8.3 to 108 mg/kg in milk chocolates. The median concentrations were 47.2 mg/kg and 18.9 mg/kg, respectively, consistent with previously reported data.^39^ In contrast, dark chocolates exhibited the lowest CML levels, with concentrations ranging from 4.3 to 18.1 mg/kg (median, 6.6 mg/kg). CML can be formed in different pathways, e.g., by direct reaction of the side-chain of lysine with GO, but also by oxidative degradation of ARPs such as fructosyllysine.^27^ The higher concentrations of CML in filled and milk chocolates may be attributed to elevated levels of ARPs, whose oxidation during the production process may lead to an increase in CML.

In this study, pyrraline levels were lowest in white chocolates (2.5–3.3 mg/kg) compared to filled (27.3–54.3 mg/kg), dark (21.9–44.4 mg/kg), and milk chocolates (9–48 mg/kg) (Figure S7). Nonparametric correlations were assessed between the concentrations of individual glycation compounds in chocolates. Here, the filled chocolates were disregarded because of the completely different methods of production and the possible carry-over from ingredients of the fillings (Table S3). A correlation analysis between different glycated amino acids can provide insight into common formation pathways of these compounds. Strong correlations indicate that certain modifications develop in parallel. Thus, the analysis provides valuable insights into the specifics of the Maillard reaction in a given food group. The best correlations are shown in Figure 4. The concentration of pyrraline correlated particularly well with the concentrations of formyline and HMF, as similarly observed in pasta and malt samples.^18,19^ Pyrraline and formyline can be formed from different sugars under the same conditions, however, they share disaccharides such as lactose as common precursors.^14^ Pyrraline and HMF are both derived from 3-DG as a precursor. There was also a strong correlation between CEL and pyrraline, leading to the hypothesis that these products also share the same precursor in chocolate, probably 3-DG, which was already suggested to occur in vivo in lens protein.^47^ Although milk chocolate showed relatively low 3-DG levels, moderate pyrraline concentrations were observed, suggesting that a portion of 3-DG may have been converted to pyrraline during processing. Filled chocolates were rich in pyrraline, most probably due to whole milk powder and condensed skimmed milk powder, as low water activity promotes pyrraline formation.^14^

**Figure 4:**
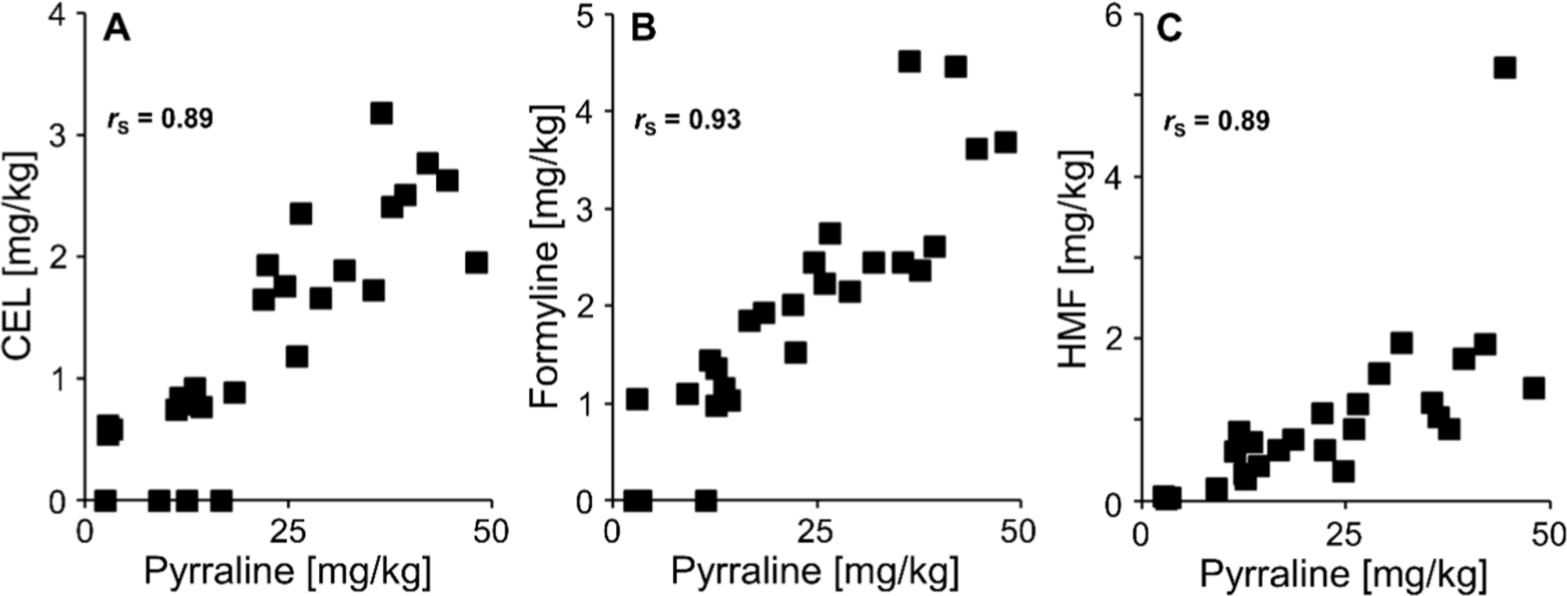
Correlations between the concentrations of pyrraline and (A) CEL, (B) formyline, and (C) HMF in all chocolates. Spearman’s rank correlation coefficients (*r*_S_) are included.

Further significant glycated amino acids found in food are MG-H1 and CEL, which are produced when MGO reacts with the side chains of arginine and lysine, respectively.^27^ CEL levels in milk (nd-2 mg/kg), white (nd-1 mg/kg), and dark chocolates (1.7–3.2 mg/kg) were lower than those in filled chocolates (2–3.4 mg/kg). MG-H1 levels were higher in milk chocolates (nd-15.8 mg/kg) than in filled (5.9–8.9 mg/kg), and dark chocolates (tr-9.1 mg/kg). In some milk and all white chocolate samples, MG-H1 was not detected. In our research, formyline levels were higher in dark (1.5–4.5 mg/kg) and filled chocolates (1.7–2.9 mg/kg) than in milk (tr–3.7 mg/kg) and white chocolates (tr–1.1 mg/kg). The low concentration of formyline can be attributed to the nondetectable levels of 3-DPs, the key precursor for formyline formation, in the analyzed samples.^14^ In contrast, maltosine and rhamnolysine were not detected in the majority of the chocolate samples.

The origin of glycated amino acids in the chocolate may be the compounds formed during roasting of cocoa, and during processing of milk powder and fillings used as ingredients, but also during the conching process. Temperature conditions, heating rate, and residence time should be the most relevant parameters for the formation of individual glycated amino acids during roasting. The particular relevance of roasting is underlined by the fact that dark chocolates, which contain the highest amount of cocoa mass, also contain the highest amounts of 3-DG, 3-DGal, HMF, CEL, formyline, maltosine, pyrraline, and rhamnolysine among the nonfilled chocolates, which are all compounds from later stages of the Maillard reaction. This is logical, because roasting is typically performed at about 120 °C for 30–60 min^38^ In milk and white chocolates, these compounds are less abundant. They are rather rich in Amadori products and CML - an oxidation product of Amadori products. Further work will have to show how individual processing steps in the production of the different chocolate groups influence the formation of individual glycation compounds, so that the process can be optimized to produce chocolates with as little protein deterioration as possible.

### Contribution of Chocolate to the Dietary Intake of Glycation Compounds

The amounts of MRPs in various chocolates, except maltosine and rhamnolysine, can be used to estimate how much MRPs are consumed through this food. Although Switzerland leads the world in chocolate consumption (approximately 32 g/day), studies on eating behavior indicate that a typical average portion of bar chocolate ranges between 25 and 60 g.^48–50^ The main differences in chocolate consumption in different countries lie not in portion size, but in the frequency of consumption. Based on consuming 50 g of chocolate per serving, Table 3 presents a summary of the individual MRPs consumed with a portion of chocolate. Consumption of chocolate greatly increases dietary exposure to MRPs. Between 48.5 and 266 mg/portion of *N*-ε-fructosyllysine can be taken up with one serving of filled chocolate, which contributes significantly to the estimated daily intake of 500–1200 mg of ARPs.^51^ Another important factor to consider is CML, for which an approximate daily consumption of 2.2–24.6 mg was found.^52,53^ The intake of CML from one portion of some filled and milk chocolates already exceeds the lower bound of reported daily intake levels.

**Table 3:** Amount (mg) of Individual Maillard Reaction Products Ingested by Consumption of a 50 g Portion of Chocolate

MRPs	types of chocolate			
	filled^a^ (*n* = 4)	dark^a^ (*n* = 12)	milk^a^ (*n* = 11)	white^a^ (*n* = 4)
3-DG	7.74 (3.8–15)	0.91 (0.52–1.43)	0.37 (0.26–0.47)	0.23 (0.08–0.35)
3-DGal	0.34 (0.23–0.44)	0.14 (0.12–0.22)	0.11 (0.03–0.19)	0.09 (0.01–0.14)
HMF	1.4 (0.73–2.3)	0.06(0.02–0.24)	0.03 (0.01–0.07)	0.003 (0.002–0.003)
CEL	0.14 (0.12–0.17)	0.11 (0.08–0.16)	0.04 (up to 0.14)	0.03 (up to 0.1)
CML	2.4 (1.2–3.2)	0.33(0.21–0.92)	0.94 (0.4–5.4)	0.50 (0.32–0.67)
formyline	0.12 (0.09–0.14)	0.12 (0.08–0.23)	0.07 (up to 0.18)	0.05 (up to 0.05)
*N*-ε-fructosyllysine	133 (48.5–266)	1 (0.37–2.9)	2 (0.95–3)	6.1 (5.8–7.8)
*N*-ε-lactulosyllysine	39 (15.6–42.2)	0.92 (up to 36.8)	44.2 (9.2–159)	32.4 (24.8–81.9)
maltosine	—	0.08 (up to 0.11)	—	—
MGH1	0.39 (0.31–0.45)	0.34 (up to 0.46)	0.76 (up to 0.79)	—
pyrraline	2 (1.4–2.7)	1.7 (1.1–2.2)	0.67 (0.45–2.4)	0.14 (0.12–0.16)
rhamnolysine	—	0.12 (up to 0.13)	—	—

a Data are given in mg/portion of chocolate, based on the median levels and the ranges given in parentheses. — refers to insignificant contributions when analytical data below the LOQ were obtained.

Higher heat treatments are necessary for the formation of other glycated amino acids examined in this work. Consequently, chocolate contributes less to the intake of maltosine (nd-0.1 mg per portion) when compared to a daily intake of 1–2 mg,^54^ formyline (0.05–0.12 mg per portion, compared to a daily intake of 2–3 mg^14^), and pyrraline (0.14–2.0 mg per portion, compared to a daily intake of 20–40 mg^14^). This also holds true for MG-H1 (0.34–0.76 mg per portion, compared to 15–29 mg daily intake^52^).

Enterocytes can absorb the amino acids CML, pyrraline, and formyline when they are bound as dipeptides, but not fructosyllysine.^15^ Due to their capacity to interact with biological targets, dietary MRPs are frequently seen as posing a health concern; however, this danger has not yet been conclusively demonstrated by trustworthy structure–activity correlations.^6,55^ Hence, eating chocolate cannot be directly linked to any health risks. However, because chocolate intake is quantitatively significant in certain regions of the world (e.g., Europe, North America), it should be included in future research on the health impacts of MRPs. Another issue is that the manufacturing of chocolate partially blocks the essential amino acid lysine, which lowers the nutritional value of the protein. This is particularly crucial for kids as they grow. On the other hand, the probiotic effects of proteins modified with either galactose or lactose, which leads to accumulation of *N*-ε-tagatosyllysine and *N*-ε-lactulosyllysine, were discussed to elicit positive probiotic effects.^56^

In conclusion, the current work used HPLC-UV and HPLC-MS techniques to examine MRPs in various chocolate varieties, including milk, dark, white, and filled chocolates. Furosine content was higher in chocolates with filling and milk chocolates, indicating early Maillard reaction stages. *N*-ε-Fructosyllysine was found to be the main ARP in the chocolates with filling, while *N*-ε-lactulosyllysine was the main ARP in the milk chocolates, and pyrraline and CML were the main glycated amino acids from the late stage of glycation. However, 3-DG, HMF and 3-DGal were lower in milk and white chocolates compared to other types of chocolates. Maltosine, MG-H1, and rhamnolysine were not detected in some of the chocolate samples. The higher levels of MRPs observed in certain chocolates are likely associated with processing steps such as roasting, fermentation, and conching, as well as the addition of sugar and protein-rich ingredients. Especially the concentrations of the Amadori product *N*-ε-lactulosyllysine may also serve as a quality marker for chocolates, as it can indicate the degree of heat damage that a milk powder utilized for chocolate production may have undergone. Due to their wide concentration ranges observed in chocolate samples, CML, *N*-ε-fructosyllysine, *N*-ε-lactulosyllysine, pyrraline, and HMF appear to be the most sensitive markers for assessing the degree of Maillard reaction during the manufacturing of chocolates. Dietary intake of *N*-ε-fructosyllysine, CML, pyrraline, and *N*-ε-lactulosyllysine is increased by the ingestion of milk and filled chocolates at common serving sizes.

## Supplementary Material



## Notes

Calibration curves utilized during evaluation of HPLC-MS/MS chromatograms (Figure S1). RP-HPLC-UV analysis of furosine in chocolate samples (Figure S2). Boxplots showing the distribution of the MRP concentrations in different chocolates (Figure S3). RP-HPLC-UV analysis of dicarbonyl compounds in chocolate samples (Figure S4). RP-HPLC with UV and time-of-flight mass-spectrometric detection of a defatted milk chocolate sample derivatized with *o*-phenylenediamine (Figure S5). RP-HPLC-UV analysis of HMF in chocolate samples (Figure S6). RP-HPLC-UV analysis of pyrraline in chocolate samples (Figure S7). List of chocolate samples with ingredients and nutritional composition (Table S1). Concentrations of individual Maillard reaction products in different chocolates (Table S2). Correlations between individual MRPs in chocolate samples (Table S3) (PDF)

## Abbreviations Used

3-DG: 3-deoxyglucosone
3-DGal: 3-deoxygalactosone
3-DPs: 3-deoxypentosone
ARP: Amadori rearrangement product
CML: *N*-ε-carboxymethyllysine
CEL: *N*-ε-carboxyethyllysine
DAD: diode array detection
GO: glyoxal
HMF: 5-hydroxymethylfurfural
HPLC: high performance liquid chromatography
LOQ: limit of quantitation
LOD: limit of detection
MG-H1: methylglyoxal-derived hydroimidazolone 1
MGO: methylglyoxal
MRM: multiple reaction monitoring
MRP: Maillard reaction product
MS: mass spectrometry
nd: not detectable
NFPA: nonafluoropentanoic acid
OPD: *o*-phenylenediamine
RP: reversed-phase
TFA: trifluoroacetic acid
tr: traces
UHPLC: ultrahigh pressure liquid chromatography
TOF: time-of-flight
UV: ultraviolet
