# Supplementary material for: Study on the Extent of the Maillard Reaction in Chocolate
Source: J Agric Food Chem. 2025 Sep 2;73(36):22724–33. doi: 10.1021/acs.jafc.5c06248 (PMC12426942; doi:10.1021/acs.jafc.5c06248)
Supplement: Supplementary file 1 [file jf5c06248_si_001.pdf]

Study on the Extent of the Maillard Reaction in Chocolate

Thirumal Sundaresan,[1,2] P. Srinivasa Rao,[2] Michael Hellwig[1,3]

[1]Chair of Special Food Chemistry, Technische Universität Dresden, D-01062 Dresden, Germany

[2]Department of Agricultural and Food Engineering, Indian Institute of Technology Kharagpur, 721302 Kharagpur, India

[3]Institute of Food Chemistry, Technische Universität Braunschweig, Schleinitzstraße 20, D-38106 Braunschweig, Germany

Corresponding author:

M. Hellwig

Technische Universität Dresden

Chair of Special Food Chemistry

Bergstraße 66

D-01062 Dresden, Germany

Tel.: +49-351-463-32006

*E-mail: Michael.Hellwig@tu-dresden.de

- Supplementary material –

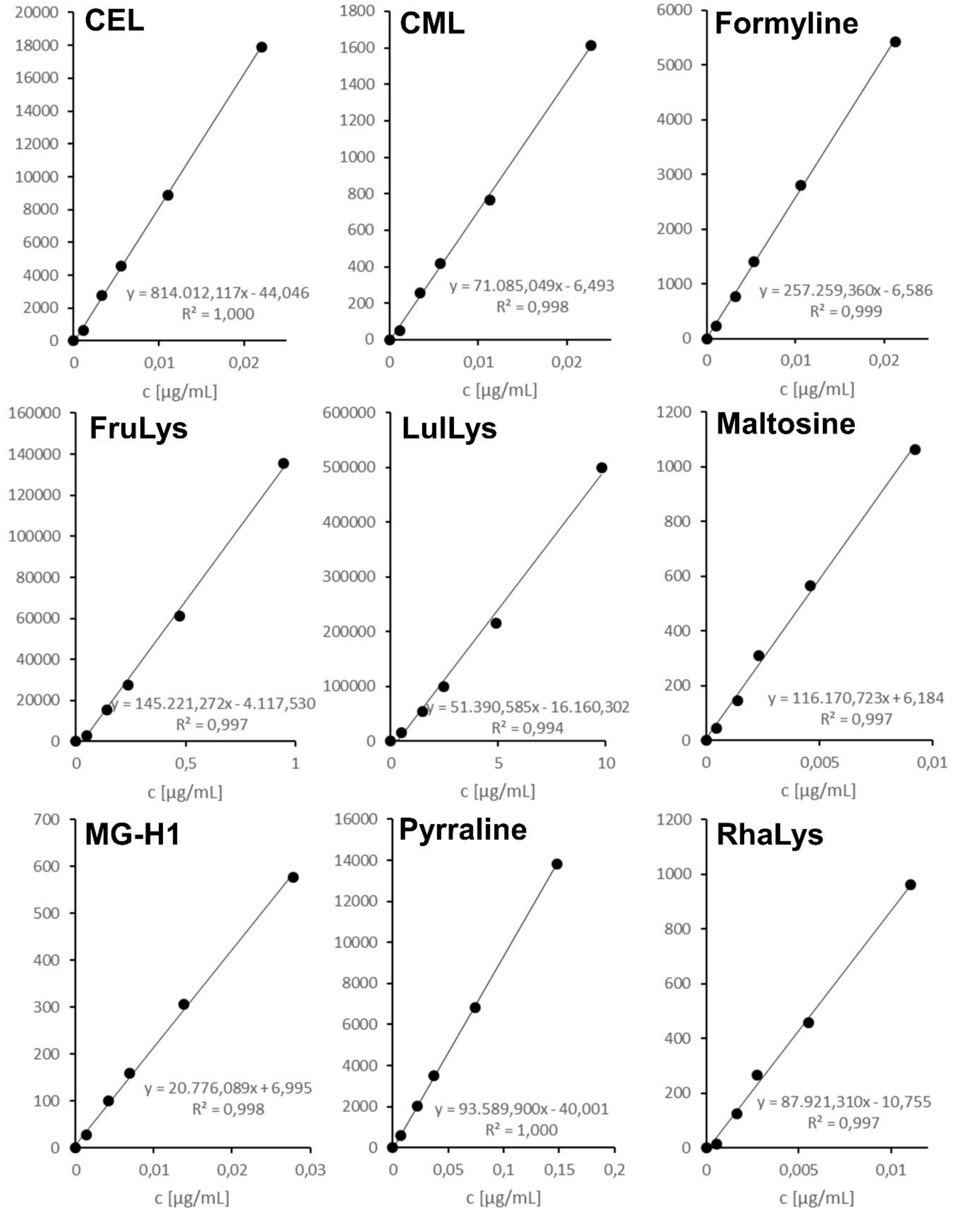

Figure S1. Matrix calibration curves used for evaluation of HPLC-MS/MS chromatograms. Peak areas of quantifier transitions are plotted against the concentration of individual MRPs added to an enzymatic hydrolysate of a defatted chocolate sample. CEL, carboxyethyllysine; CML, carboxymethyllysine; Fom, formyline; FruLys, fructosyllysine; Fur, furosine; LulLys, lactulosyllysine; MG-H1, methylglyoxal-derived hydroimidazolone 1; Pyrr, pyrraline.

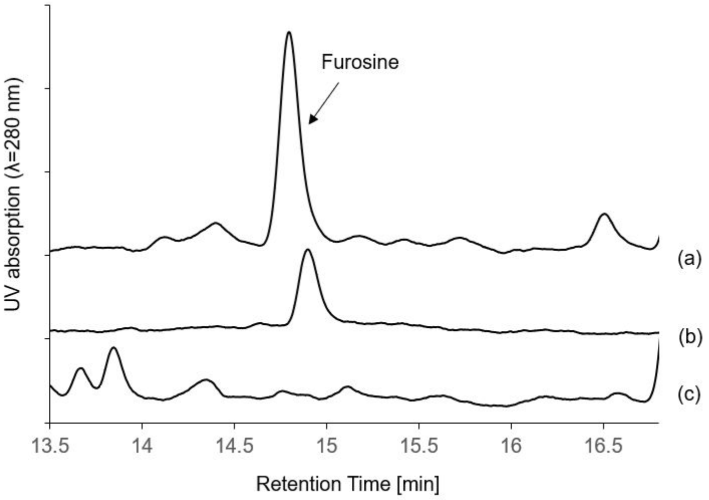

Fig. S2 RP-HPLC with UV detection. Chromatograms of (a) a chocolate sample with a high furosine content, (b) a furosine standard, and (c) a chocolate sample with a low furosine content.

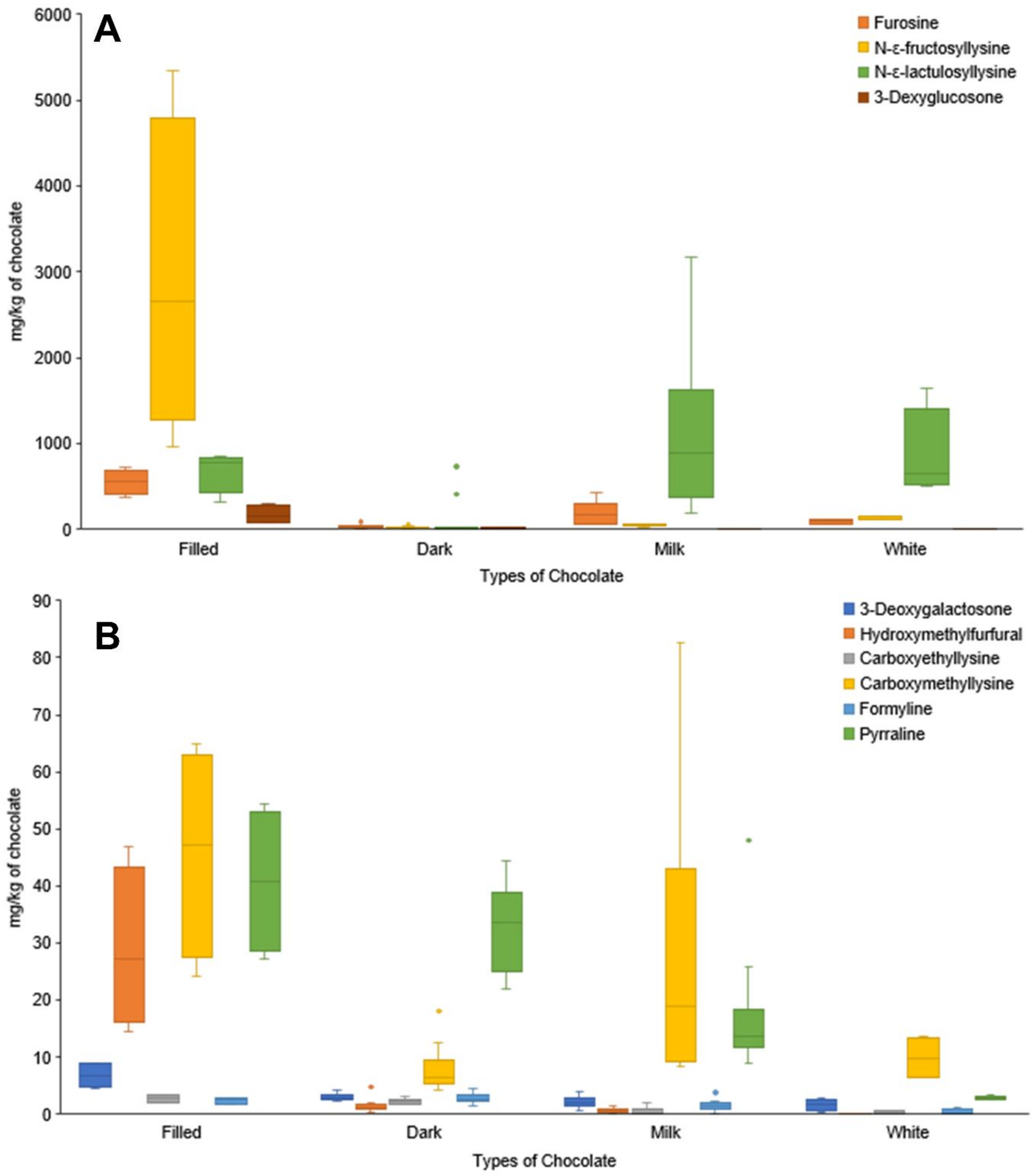

Fig. S3 Boxplots showing the distribution of the concentrations of individual MRPs in different types of chocolate. The box represents the interquartile range (25th-75th percentile), with the horizontal line indicating the median. The whiskers extend to the smallest and largest values within 1.5 × interquartile range below and above the box. Data points outside this range are displayed as individual outliers.

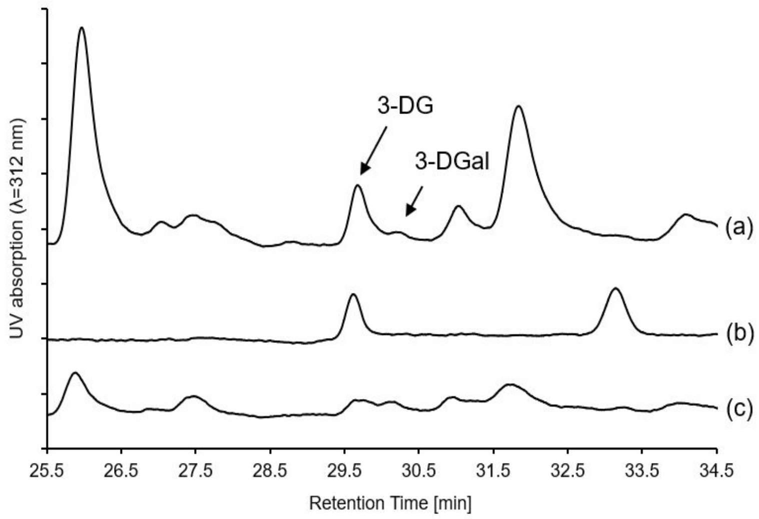

Fig. S4 RP-HPLC with UV detection. Chromatograms of (a) a chocolate sample with a high dicarbonyl content, (b) a dicarbonyl standard, and (c) a chocolate sample with a low dicarbonyl content.

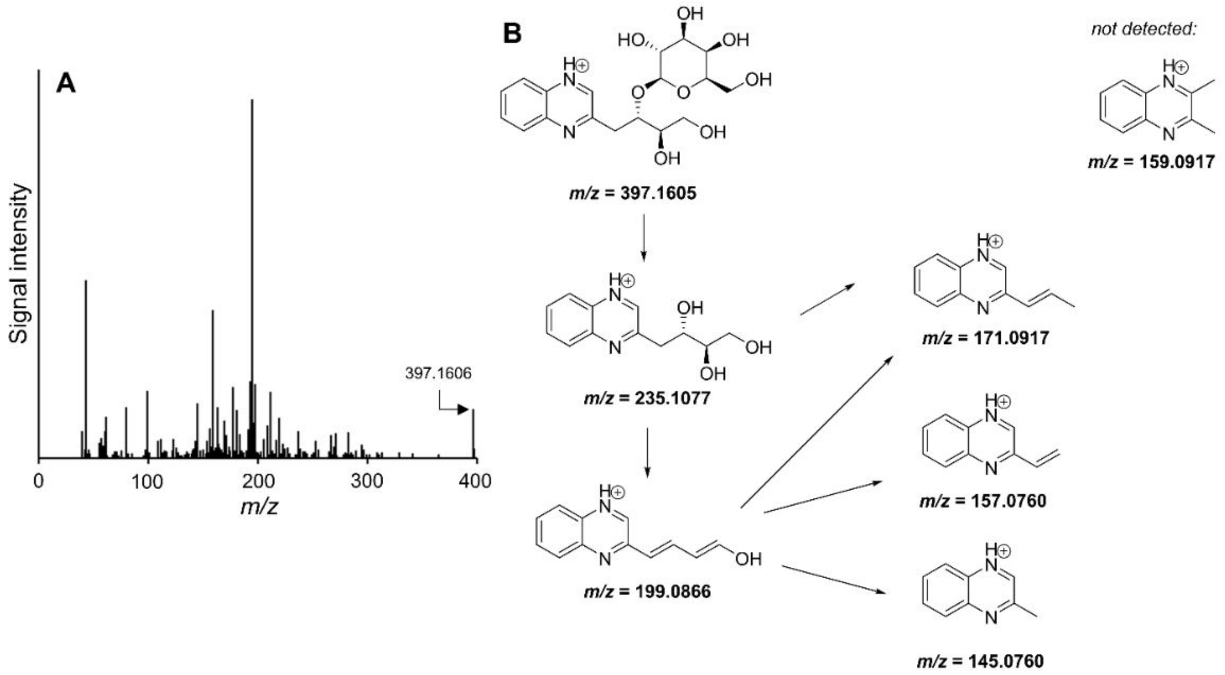

Fig. S5: RP-HPLC with UV and mass-spectrometric detection of a defatted milk chocolate sample derivatized with *o*-phenylenediamine. (A) MS/MS spectrum of peak Q1. (B) Proposed fragmentation of the quinoxaline of 3-deoxylactosone.

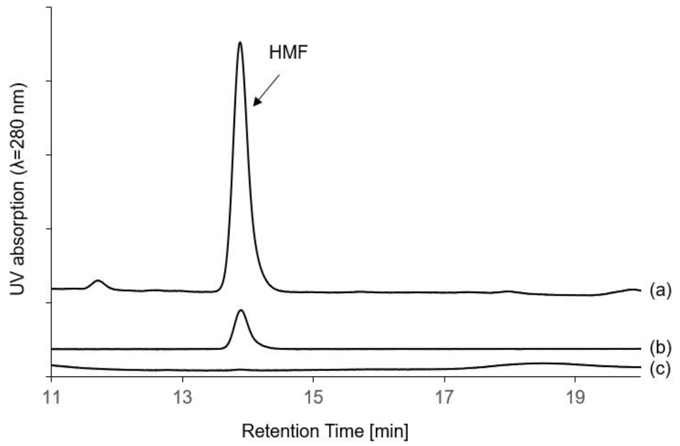

Fig. S6 RP-HPLC with UV detection. Chromatograms of (a) a chocolate sample with a high HMF content, (b) a HMF standard, and (c) a chocolate sample with a low HMF content.

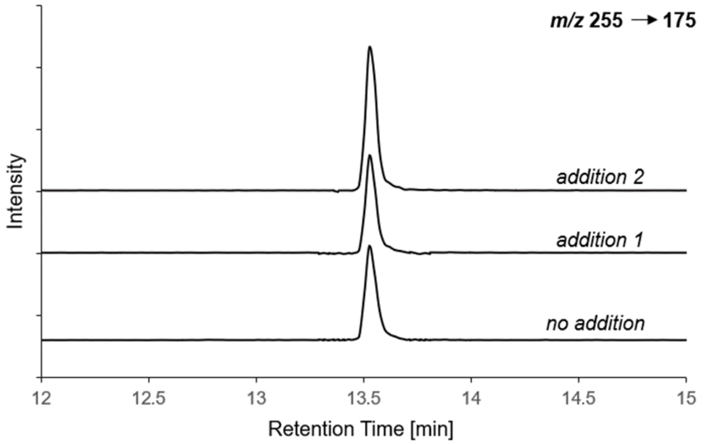

Fig. S7 RP-HPLC-MS (MRM mode) of enzymatically hydrolysed chocolate sample without and with pyrraline standard added.

Table S1. Ingredient and Nutritional Composition of Different Types of Chocolate Samples.

Sample Code	Ingredients	Protein[a]	Fat[a]	Saturated Fat[a]	Carbohydrate[a]	Sugar[a]	Dietary Fiber[a]	Cocoa[b]
D1	Sugar, cocoa mass, skimmed milk powder (11%), cocoa butter, butter fat (6%)	7.3	33	20	51	49	5.3	46% at least
D2	Sugar, cocoa mass, cocoa butter, pure butter fat	6.8	31	19	52	47	-	50% at least
D3	Sugar, cocoa mass, cocoa butter	6.0	33	19	50	48	-	50% at least
D4	Sugar, cocoa mass, cocoa butter, skimmed milk powder, butter purified fat, sweet whey powder (milk)	7.6	36	22	47	44	5.5	45% at least
D5	Cocoa mass, sugar, cocoa butter	5.6	30.1	18.7	51.1	47.8	-	50% at least
D6	Sugar, cocoa mass, cocoa butter	4.6	32	19	55	49	7	50% at least
D7	Cocoa mass, sugar, cocoa butter, low fat cocoa powder	8.3	49	29	26	18	12	80% at least
D8	Cocoa mass, cocoa butter, cane sugar, ground vanilla pods	7.8	54	33	24	14	12	85% at least
D9	Sugar, cocoa mass, cocoa butter	6.4	40.3	24.7	41	34.9	8.4	63% at least
D10	Sugar, cocoa mass, cocoa butter	8.2	56.7	34.5	19.8	12.6	10.7	85% at least
D11	Sugar, cocoa mass, cocoa butter	9.5	42.6	25.7	33.5	27.9	-	72% at least
D12f	Sugar, whole milk powder (24%), cocoa mass, hazelnut bits(6%), glucose syrup, cocoa butter, dextrose, butter, condensed skimmed milk	9.0	20.4	10.3	59.9	56	2.5	75% at least
D13	Sugar, cocoa mass, cocoa butter, milk fat	5.9	29	19	51	49	-	50% at least

Sample Code	Ingredients	Protein[a]	Fat[a]	Saturated Fat[a]	Carbohydrate[a]	Sugar[a]	Dietary Fiber[a]	Cocoa[b]
M1	Sugar, cocoa butter, hazelnut mass (13%), cocoa mass, skimmed milk powder, sweet whey powder (from milk), butter purified fat	7.6	35	17	51	49	3.2	33% at least
M2	Sugar, cocoa butter, cocoa mass, skimmed milk powder, cocoa mass, sweetener powder, butter fat, hazelnut mass	6.5	31	19	57	55	2.3	33% at least
M3	Sugar, cocoa butter, cocoa mass, whole milk powder (10%), skimmed milk powder, butterfat	6.6	30	19	58	57	-	30% at least
M4	Sugar, cocoa butter, cream powder (13%), cocoa mass, whole milk powder (5.4%)	5.5	33	20	57	56	-	30% at least
M5	Sugar, cocoa butter, whole milk powder (16%), sweet whey powder, cocoa mass, hazelnut paste, butter fat	6.3	31.2	18.5	57.1	56.5	-	30% at least
M6	Sugar, cocoa butter, whole milk powder (21%), cocoa mass	7.3	38	23	48	47	-	35% at least
M7	Sugar (41%), whole milk powder, cocoa butter, cocoa mass, ground hazelnuts	7.7	35	21	53	52	-	31% at least
M8	Cane sugar, cocoa butter, whole milk powder, cocoa mass, skimmed milk powder, vanilla pods	7.9	38	23	50	48	2.2	37% at least
M9	Sugar, cocoa butter, whole milk powder(23.5%), cocoa mass, clarified butter, vanilla extract	7.1	38.2	23.8	50.6	49.8	-	32% at least
M10f	Sugar, whole milk powder (28%), cocoa mass, hazelnut bits (6%), glucose syrup, cocoa butter, dextrose, butter, condensed skimmed milk	8.6	20.6	10.1	61.4	52.1	2.8	30% at least

Sample Code	Ingredients	Protein[a]	Fat[a]	Saturated Fat[a]	Carbohydrate[a]	Sugar[a]	Dietary Fiber[a]	Cocoa[b]
M11f	Sugar, whole milk powder (31%), cocoa butter, hazelnut bits (6%), glucose syrup, egg liqueur (3%), cocoa mass, butter, condensed skimmed milk	8.8	20.5	11.2	61.1	50.9	1.3	30% at least
M12	Sugar, cocoa butter, hazelnut (14%), cocoa mass, whole milk powder (2.4%), sweet whey powder, skimmed milk powder, butterfat	6.3	35	17	53	52	-	30% at least
M13	Sugar, cocoa butter, cocoa mass, whole milk powder (6.6%), skimmed milk powder (6.5%), whey powder, milk fat	5.9	29	19	59	59	-	30% at least
W1	Sugar, cocoa butter, whole milk powder, cream powder, lactose, sweet whey powder	6.3	38.7	23.7	52	52	-	29% at least
W2	Sugar, cocoa butter, skimmed milk powder, sweet whey powder (from milk), butterfat	4.2	28	17	65	65	0	-
W3	Sugar, cocoa butter, skimmed milk powder, whole milk powder, milk sugar, butter purified fat	5.4	33	20	58	58	-	-
W4f	Sugar, whole milk powder (28%), cocoa butter, hazelnut bits (6%), glucose syrup, dextrose, skimmed milk powder, butter, condensed skimmed milk	9.0	20.9	10.8	62.1	59.2	0.5	30% at least
W5	Sugar, cocoa butter, whole milk powder, milk sugar, whey powder, cream powder	6.4	35	22	56	56	-	-

[a] Data are given in g/100 g.

[b] Data are given in percentage.

D12, M10, M11, and W4 denotes filled chocolates. These are also marked with an added f.

Table S2. Concentrations of Individual Maillard Reaction Products in Different Varieties of Chocolate Samples.

Sample Code	Furosine[a]	3-DG[a]	3-DGal[a]	HMF[a]	CEL[a]	CML[a]	Formyline[a]	*N-ε-*fructosyl lysine[a]	*N-ε-*lactulosyl lysine[a]	Maltosine[a]	MGH1[a]	Pyrraline[a]	Rhamnolysine[a]
D1	84.1	12.3	2.3	0.37	1.8	12.4	2.5	43.4	407	tr	6.2	24.5	nd
D2	24.8	20.0	2.9	1.5	1.7	7.6	2.1	28.6	4.3	nd	tr	28.9	nd
D3	9.2	14.9	3.4	0.66	1.9	5.7	1.5	19.9	7.8	nd	tr	22.4	nd
D4	103	18.4	3.7	1.1	1.7	18.1	2.0	57.5	736	nd	tr	21.9	nd
D5	15.5	20.7	2.8	0.87	2.4	6.8	2.4	23.3	27.5	tr	6.4	37.6	nd
D6	17.2	12.2	2.3	1.8	1.9	4.3	2.5	14.1	tr	nd	6.8	31.8	nd
D7	38.3	17.4	2.7	1.3	2.4	6.3	2.8	16.8	18.5	tr	8.4	26.5	nd
D8	36.5	28.6	4.3	1.2	1.7	4.9	2.5	26.6	4.1	1.5	5.7	35.4	2.7
D9	32.4	20.6	2.5	4.8	2.6	8.5	3.6	19.2	nd	tr	6.8	44.4	tr
D10	13.9	20.3	2.5	1.9	2.8	9.7	4.5	21.8	nd	1.3	8.4	41.9	2.1
D11	28.4	18.0	3.1	1.5	2.5	5.8	2.6	7.3	nd	1.5	6.9	39.3	nd
D12f	369	76.0	4.6	21.5	2.3	24.2	2.1	969	312	nd	6.9	27.3	nd
D13	7.4	10.4	3.4	0.99	3.2	5.3	4.5	12.9	nd	1.9	9.1	36.3	tr
M1	66.2	9.1	2.7	0.85	1.2	9.1	2.2	59.0	388	nd	15.7	25.9	nd
M2	134	9.4	3.4	0.63	0.75	8.3	tr	50.4	351	nd	tr	11.3	nd
M3	293	7.4	2.5	0.69	0.89	25.4	1.9	53.6	1624	nd	nd	18.4	nd

Sample Code	Furosine[a]	3-DG[a]	3-DGal[a]	HMF[a]	CEL[a]	CML[a]	Formyline[a]	*N-ε*-fructosyl lysine[a]	*N-ε*-lactulosyl lysine[a]	Maltosine[a]	MGH1[a]	Pyrraline[a]	Rhamnolysine[a]
M4	55.1	6.3	1.1	0.32	0.79	8.3	0.98	38.6	377	nd	nd	12.5	nd
M5	169	6.6	1.7	0.74	0.92	18.9	1.2	54.4	1408	nd	tr	13.5	nd
M6	82.3	8.3	1.5	0.46	0.77	9.2	1.0	38.5	512	nd	nd	14.2	nd
M7	421	5.2	2.6	0.63	tr	108	1.9	40.7	3170	nd	tr	16.6	nd
M8	300	8.5	2.1	0.14	nd	82.5	1.1	30.9	2138	nd	nd	9.0	nd
M9	177	6.0	0.57	0.34	tr	42.3	1.4	37.2	1137	nd	tr	12.5	nd
M10f	596	192	8.9	32.9	3.1	57.5	2.9	2184	805	nd	8.5	49.4	nd
M11f	714	299	8.5	14.5	3.4	64.8	2.8	3142	768	nd	8.9	54.3	nd
M12	174	7.1	3.9	1.4	1.9	42.9	3.7	42.5	884	tr	14.8	48.0	nd
M13	53.3	7.5	2.0	0.83	0.85	11.5	1.4	19.2	184	nd	tr	11.7	nd
W1	121	5.2	2.8	0.05	0.59	13.5	tr	156	1637	nd	nd	3.3	nd
W2	55.0	4.1	1.9	0.05	0.62	6.4	tr	116	496	nd	nd	2.8	nd
W3	70.5	1.6	0.28	0.04	0.54	6.8	1.1	122	671	nd	nd	2.8	nd
W4f	517	118	5.1	46.8	2.0	37.0	1.7	5337	843	nd	5.9	32.2	nd
W5	106	6.9	1.6	0.06	nd	12.8	tr	123	627	nd	nd	2.5	nd

[a] Data are given in mg/kg of chocolate. nd, not detectable, below LOQ; tr, traces, between LOD and LOQ. D12, M10, M11, and W4 denotes filled chocolates. These are also marked with an added f.

Table S3. Correlations between individual MRPs in chocolate samples ($n = 27$; filled chocolates omitted). Spearman's rank correlation coefficients (r_S) and significance of correlations (P) were calculated using the software PASW Statistics 18. Only significant correlations ($P < 0.01$) with $r_S > 0.5$ are reported. 3-DG, 3-deoxyglucosone; 3-DGal, 3-deoxygalactosone; CEL, carboxyethyllysine; CML, carboxymethyllysine; Fom, formyline; FruLys, fructosyllysine; Fur, furosine; HMF, 5-hydroxymethylfurfural; LulLys, lactulosyllysine; MG-H1, methylglyoxal-derived hydroimidazolone 1; Pyrr, pyrraline.

		CML	Fom	FruLys	LulLys	MGH1	Pyrr	Fur	3-DG	3-DGal	HMF
CEL	rS	-0,501	0,862	-0,652	-0,763	0,77	0,891	-0,734	0,767	0,544	0,816
	P	,008	,000	,000	,000	,000	,000	,000	,000	,003	,000
	N	27	27	27	27	27	27	27	27	27	27
CML	rS				0,806			0,835			
	P				,000			,000			
	N				27			27			
Fom	rS			-0,638	-0,569	0,843	0,929		0,717		0,829
	P			,000	,002	,000	,000		,000		,000
	N			27	27	27	27		27		27
FruLys	rS				0,727		-0,635	0,643	-0,65		-0,664
	P				,000		,000	,000	,000		,000
	N				27		27	27	27		27
LulLys	rS					-0,561	-0,615	0,911	-0,727		-0,669
	P					,002	,001	,000	,000		,000
	N					27	27	27	27		27
MGH1	rS						0,799		0,583		0,665
	P						,000		,001		,000
	N						27		27		27
Pyrr	rS							-0,528	0,765	0,579	0,893
	P							,005	,000	,002	,000
	N							27	27	27	27
Fur	rS								-0,616		-0,529
	P								,001		,005
	N								27		27
3-DG	rS									0,57	0,779
	P									,002	,000
	N									27	27
3-DGal	rS										0,548
	P										,003
	N										27
